# Raw water biofiltration for surface water manganese control

**DOI:** 10.1038/s41598-023-36348-1

**Published:** 2023-06-03

**Authors:** Martin R. Earle, Amina K. Stoddart, Graham A. Gagnon

**Affiliations:** Centre for Water Resources Studies, Department of Civil and Resource Engineering, Dalhousie University, Halifax, NS Canada

**Keywords:** Civil engineering, Environmental sciences

## Abstract

Manganese (Mn) control in surface water systems is a challenge for the drinking water industry, especially through a sustainability framework. Current methods for removing manganese from surface water use strong oxidants that embed carbon and can be expensive and harmful to human health and the environment. In this study, we used a simple biofilter design to remove manganese from lake water, without conventional surface water pre-treatments. Biofilters with aerated influent removed manganese to concentrations below 10 µg/L when receiving influent water containing > 120 µg/L of dissolved manganese. Manganese removal was not inhibited by high iron loadings or poor ammonia removal, suggesting that removal mechanisms may differ from groundwater biofilters. Experimental biofilters also achieved lower effluent manganese concentrations than the full-scale conventional treatment process, while receiving higher manganese concentrations. This biological approach could help achieve sustainable development goals.

## Introduction

Manganese (Mn) in drinking water is typically removed to improve water aesthetics, because particulate manganese can discolour water and stain fixtures and laundry^1^. However, there are increasing concerns that high manganese concentrations may impact the health and development of young children^2–5^. Both aesthetic and health concerns can be compounded when manganese accumulates in distribution systems. Accumulated manganese can rapidly mobilize due to hydraulic or water quality changes in a distribution system, causing a large and difficult to predict spike in manganese concentration at the tap^6^. Accumulation of manganese may also increase lead release^7,8^. Therefore, manganese removal at drinking water treatment plants must be maximized.

Health Canada recommends an aesthetic objective of 20 µg Mn/L, but many utilities target lower concentrations to prevent the impacts of accumulation in distribution systems^6,9^. For surface water treatment plants, these objectives are commonly achieved using a combination of chemical oxidation using strong oxidants (e.g., chlorine dioxide or permanganate) followed by particle destabilization, or catalytic oxidation with free chlorine and manganese-oxide coated media^6,10^. However, these chemical-based techniques may produce harmful byproducts and increase treatment costs due to chemical demand, equipment required for dosing, and operator training^11,12^.

These conventional approaches to manganese treatment are unsustainable and are not practical in areas without access to specialized equipment, training, and water treatment chemicals. Their use conflicts with the United Nations Sustainable Development Goal 6 (SDG6), which intends to “ensure availability and sustainable management of water and sanitation for all”^13^. Alternative treatment technologies for manganese are required to achieve these goals. Such technologies must be capable of removing manganese to concentrations below 20 µg/L while reducing the environmental impact of drinking water treatment.

Biofiltration is a sustainable technology alternative for manganese treatment that requires little to no chemical addition, depending on influent water quality. Biofilters are granular media filters operated with low or no oxidant residual (i.e., chlorine), which allows naturally occurring microorganisms to grow and develop biofilms that coat the media^14^. These biofilms can increase the removal capacity of a filter by adsorbing and degrading dissolved contaminants, which would not be retained otherwise. Biofiltration is used extensively to treat groundwater, with many treatment systems only composed of aeration and granular media filtration^15^. Surface water treatment could be more sustainable if this clean technology was used, but the studies considering surface water biofiltration for manganese have been unable to provide widely applicable design guidance. The findings of groundwater studies are promising, but may not be directly applicable to surface water treatment because most groundwaters contain primarily dissolved contaminants and low concentrations of organic carbon, compared to surface waters. Therefore, to help achieve SDG6, surface water biofilters must be studied to establish their manganese removal capacity and design principles.

Most surface water studies have considered biofilters in a conventional surface water context, with the biofilter receiving water that has been pretreated with oxidants and has been clarified. The focus of these studies has been reducing the time to effective manganese control (i.e., acclimation time), which can be in the order of several months^16–20^. In this study, we instead propose using the conventional groundwater biofiltration approach to treat surface water. Our objective was to determine if raw water biofiltration could be an effective barrier for dissolved manganese in surface water. Using a simple aeration-biofiltration design, four conditions were studied for approximately 300 days to demonstrate the impact of dissolved oxygen, ammonia, and iron on manganese removal from surface water, with concentrations of manganese and iron exceeding 1.5 mg/L at times. A previous study considered direct surface water biofiltration, but it was focused on the effect of nutrient and peroxide addition at a smaller scale and did not consider the effect of dissolved oxygen on manganese removal^19^.

## Methods

### Biofilter design and source water

**Figure 1:**
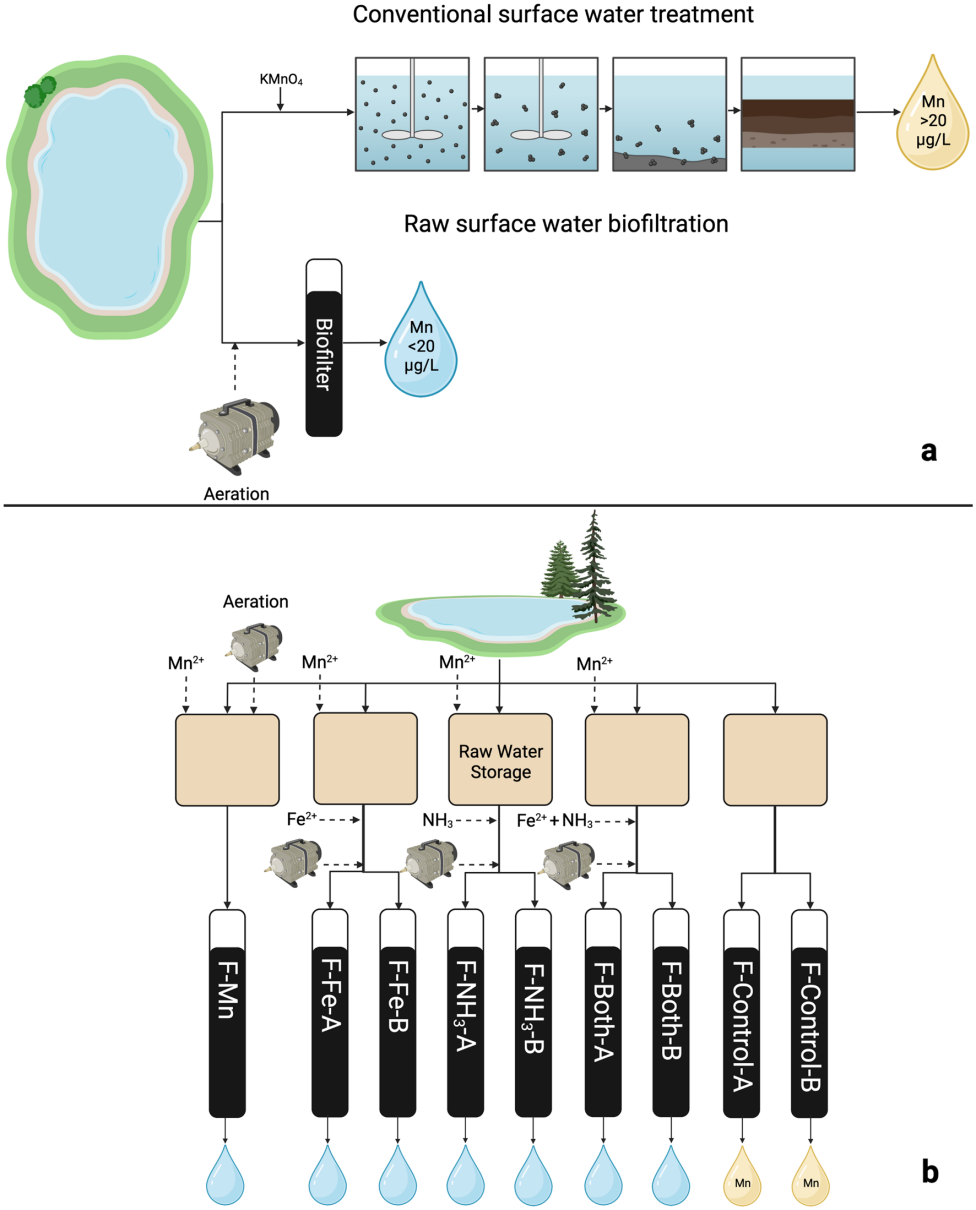
Conceptual diagram of raw water biofiltration experiment compared to the conventional surface water treatment plant hosting the experiment (**a**) and diagram of raw water biofiltration experimental conditions.

Biofiltration experiments were hosted at the Bennery Lake Drinking Water Treatment Plant (BLDWTP) in Nova Scotia, Canada, which utilizes conventional surface water treatment (Fig. 1a). Bennery Lake was chosen for this study because seasonal stratification leads to exceptional manganese and iron (Fe) concentrations, more than 1.5 and 2.0 mg/L respectively. The lake stratifies in early summer, causing the eventual depletion of dissolved oxygen in the hypolimnion. Without a supply of oxygen, the sediments of Bennery Lake dissolve, leading to a steady increase in manganese and iron concentration until the lake destratifies in early fall. After destratification, dissolved oxygen concentrations increase, causing dissolved manganese and iron to oxidize and precipitate and return to the sediment. Bennery Lake destratified on day 107 of this experiment. Organic carbon concentrations are moderate to high in this lake, with the total organic carbon (TOC) typically below 6.0 mg/L. However, TOC concentrations approaching 9.0 mg/L were observed at times. Alkalinity and hardness are both low in Bennery lake at approximately 5.0 and 8.0 mg/L as CaCO_3_, respectively^21^.

Biofilters were contained within 2.5 cm diameter glass columns that were 200 cm tall (FlexColumn™ Economy Columns, DWK Life Sciences). They were filled with approximately 61 cm of fresh anthracite (effective size = 0.9 mm) over 30 cm of fresh sand (effective size = 0.45 mm), matching the full-scale media profile of BLDWTP. Nine filters were operated for the duration of the study (286 days) with four conditions (Fig. 1b). Two control filters were fed unmodified raw water. Experimental filters were fed additional dissolved Mn (0.1–0.5 mg/L), with the remaining conditions being additional iron, ammonia and combined iron and ammonia at concentrations of 0.1 mg/L. All biofilters were operated at a flowrate of approximately 28 mL/min (3.3 m/h) for most of the study and flowrates were calibrated regularly. This led to an empty bed contact time of 16 min or less, due to a reduction in filter bed volume over time as filter media was removed for sampling. Flowrates were decreased to approximately 14 mL/min (1.7 m/h) from days 192 to 256 because access to BLDWTP was limited due to local COVID-19 related restrictions beginning in December 2021. During this time manganese and ammonia dosing remained consistent, but iron dosing was stopped and was not resumed. Sampling also could not be completed during this time. Biofilters were generally backwashed weekly with either untreated lake water or chlorine-free full-scale treated water at a flowrate of approximately 150 mL/min until the backwash water was visibly clear. Backwashing was reduced to twice per month during the period of reduced flowrates. During backwashes, a stainless-steel rod was used to disrupt media and release air pockets formed while washing.

BLWDTP produced water for 6 to 7 h each day, therefore, raw water was stored in 200 L plastic reservoirs to enable continuous flow across pilot biofilters. All filters except for controls had dissolved manganese added to the storage reservoirs using an acidified stock solution (1 or 10 g Mn(II)/L, Mn(II)SO_4_·H_2_O, Fisher Scientific). Sodium bicarbonate (Fisher Scientific) was added directly to the storage reservoirs to offset alkalinity loss from the acidified stock solution. Diluted iron and ammonia stock solutions were stored in smaller containers (15–40 L) and pumped to an intermediate mixing vessel (5 L) with the modified raw water to produce the biofilter influent water. These dilute stock solutions were made from concentrated ammonium (1 g NH_4_ as N/L, NH_4_Cl, Fisher Scientific), iron (1 g Fe(II)/L, FeSO_4_·7H_2_O, Fisher Scientific), and manganese stock solutions and untreated lake water. Dilute solutions containing iron were acidified to below pH 4 using 1N HCl (Fisher Scientific) to prevent rapid oxidation by oxygen prior to biofiltration. Biofilter influent water for experimental filters was aerated from days 63 to 130 using an aquarium air pump, after which the raw water dissolved oxygen concentration was near saturation. The storage reservoir for filter F-Mn was aerated directly because there was no intermediate mixing vessel for filters not fed iron or ammonia.

### Water sampling and analysis

Water quality measurements were taken twice per week, except for total organic carbon (TOC), which was measured twice per month. Effluent samples were taken first by allowing effluent water to drip into glassware directly from the filter (i.e., without touching tubing). Influent samples were collected in glassware directly from the filter influent tube. After on-site analysis was completed, the water was transported in clean and HNO_3_ (Fisher Scientific) soaked bottles to the Dalhousie Clean Water Laboratory for additional analysis.

Water pH (8157BNUMD, Orion), temperature, and dissolved oxygen concentration (083005MD, Orion) were measured on-site immediately after sampling. Dissolved oxygen measurements started on day 53. The concentration of manganese, iron, ammonia, and TOC were measured at the Dalhousie Clean Water Laboratory. Manganese and iron were measured using inductively coupled plasma-mass spectrometry (ICP-MS, iCAP™ RQ, Thermo Fisher). Samples were split and half of the volume was passed through conditioned 0.45 µm cellulose nitrate membrane filters with a syringe filter cartridge. All manganese and iron samples were preserved in new polypropylene tubes by acidifying with trace metal grade HNO_3_ (Fisher Scientific) to pH < 2 before analysis.

Ammonia was measured using UV–vis spectrophotometry (DR5000™ or DR6000™, HACH). The salicylate method was most often used (0.01–0.50 mg NH_3_-N/L, Method 8155, HACH) but a shortage of reagents resulted in the use of the ultra low range TNT method (0.02 to 2.5 mg NH_3_-N/L, TNT 830, HACH). For TOC analysis, samples were preserved by acidifying with H_3_PO_4_ (Fisher Scientific) to pH < 2 and analysed with a TOC analyser (TOC-VCPH, Shimadzu).

Asymmetric flow field-flow fractionation (FFF) was used evaluate colloidal particles (i.e., smaller than 0.45 µm) in untreated Bennery Lake water, using the methods described by Trueman et al.^22^. In short, samples were first filtered through primed 0.45 µm cellulose nitrate membrane filters, then fractionated using an asymmetric flow FFF system (AF2000 Multiflow, PostNova) with a 300 Da polyethersulfone membrane and a manual injection valve with a 1 mL polyether ether ketone sample loop. The fractionated particles were then analysed first by a UV–vis detector (SPD-20A, Shimadzu), followed by ICP-MS.

### Filter Media Sampling and Analysis

Filter media was sampled once every two weeks. Autoclave-sterilized stainless-steel scoopulas were used to first homogenize the top few inches of filter media and then to collect a 1–4 g sample. The media sample was stored in sterile 50 mL plastic tubes (Falcon®, VWR™) and transported on ice to the Dalhousie Clean Water Laboratory for immediate processing. Adenosine triphosphate (ATP) was measured immediately using a commercial test kit (Deposit & Surface Analysis, LuminUltra®) and luminometer (PhotonMaster™), following manufacturer instructions. Additional media was then prepared for metals analysis by oven drying at 105 °C or air drying in a desiccator cabinet at room temperature. Metals were extracted via heated acid digestion, following EPA Method 3050B^23^ and were measured by ICP-MS.

### Statistical analysis and visualization

All statistical analysis was conducted in the R programming environment through the RStudio IDE using the base R package version 4.0.2^24^. A significance level of α = 0.05 was used for both Spearman correlations and Wilcoxon Rank Sum tests. It was assumed that the measurements of each parameter were independent from one another. *p*-values were not reported for correlations calculated across the entire dataset because observations are likely correlated with past observations, which may result in unrealistically low *p*-values^25^. Data visualization and computation of empirical cumulative distribution functions were also completed in R using the Tidyverse package suite version 1.3.0^26^. Standard deviations displayed on figures were calculated for each group (represented by colour) for a given time period. The period was 1 day for all figures except S6, where the period was 1 week. Conceptual diagram (Fig. 1) was created with Biorender.com.

## Results and discussion

### Raw water and influent water conditions

**Figure 2:**
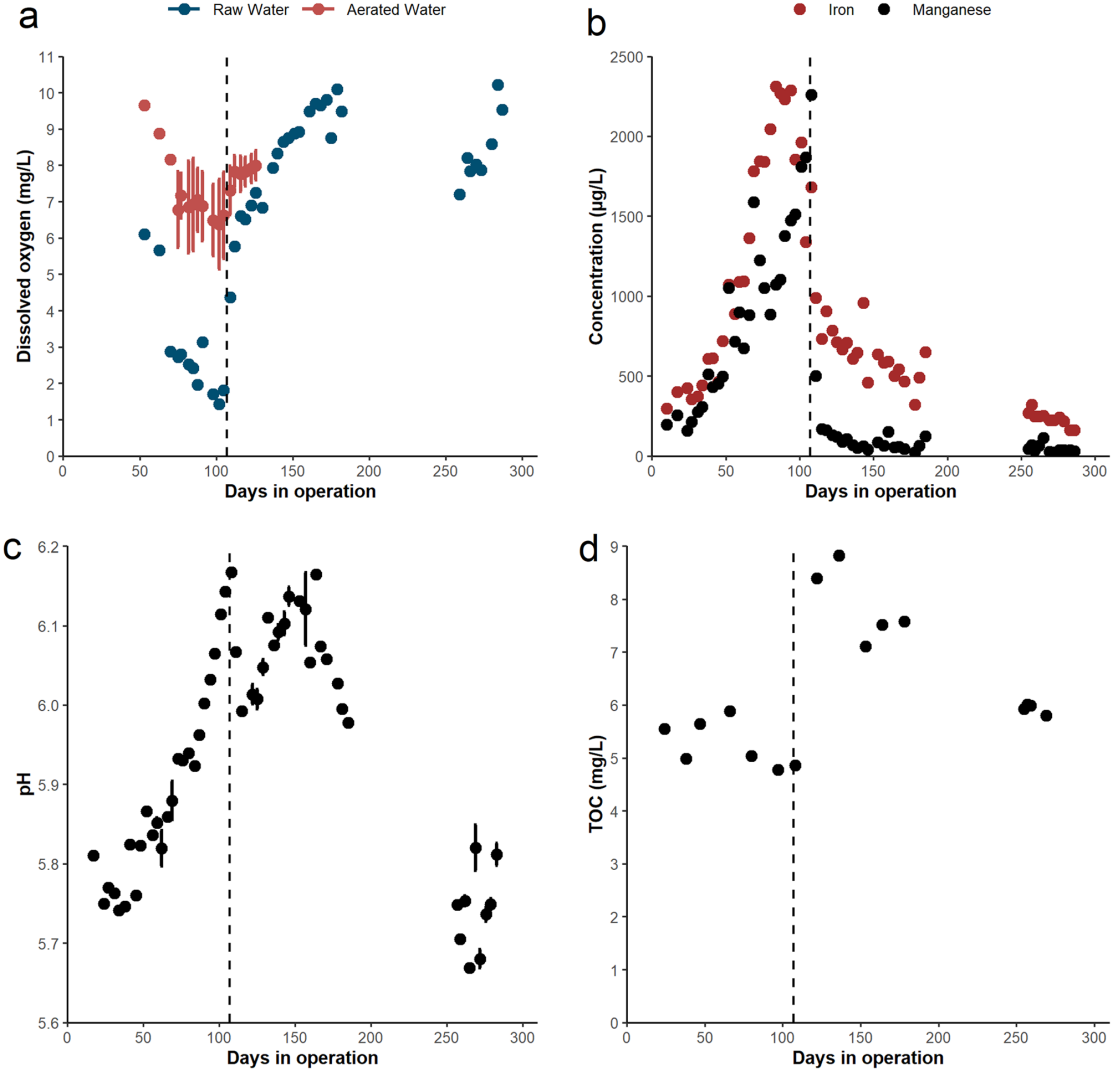
Raw water (i.e., water feeding the control filters) dissolved oxygen concentration (**a**), unfiltered manganese and iron concentrations (**b**), pH (**c**) and TOC concentration (**d**) over time. Error bars represent standard deviation. Dashed lines represent the division between lake stratification and destratification. The gap in data represents the period of COVID interruption.

Influent water quality varied over the course of the study and included three phases: lake stratification (days 0 to 107), lake destratification (days 107 to 256), and post-COVID interruption (days 256 to 285). Prior to destratification, the dissolved oxygen concentration in the raw water (i.e., water feeding the control filters) consistently decreased, to a minimum of 1.4 mg/L (Fig. 2a). This drop in oxygen resulted in a significant increase in raw water total iron and manganese concentration, both peaking at 2300 µg/L (Fig. 2b). Leading up to destratification, the proportion of iron passing a 0.45 µm filter decreased such that more than 50% of iron was particulate, suggesting that iron oxidized and conglomerated in the storage reservoirs (Supplementary Fig. S1). After destratification and the corresponding decrease in iron concentration, the particulate portion decreased to approximately 25% of the total concentration. This trend was not observed for manganese concentrations, which were mostly dissolved prior to destratification and then 30–70% particulate (Supplementary Fig. S1). The average raw water ammonia concentration was 0.05 ± 0.02 mg/L, peaking at 0.1 mg/L before destratification and dropping to below 0.04 mg/L after. The average influent water temperature was 17 ± 2 °C, however, water was colder for several hours after the storage reservoirs were refilled when the lake temperature decreased (Supplementary Fig. S2). The pH steadily increased from 5.8 to 6.1 before destratification (Fig. 2c), while the TOC concentration sharply increased from 5 mg/L to over 8 mg/L after destratification, but slowly decreased back to near 6 mg/L (Fig. 2d). When sampling resumed after local COVID-19 restrictions ended, the influent water quality conditions had mostly stabilized. Iron concentrations were below 500 µg/L, while manganese was typically below 70 µg/L. The pH had reduced to between 5.7 and 5.9, but the dissolved oxygen concentration was initially lower and increased over time due to temporary ice coverage.

Influent water for the experimental filters was adjusted for manganese, iron, ammonia, and dissolved oxygen. While the lake was stratified, iron and manganese concentrations were slightly increased in these filters. However, after the lake destratified the manganese dose was increased to compensate for the sudden decrease in raw water dissolved manganese concentration, from a raw water concentration of 36 ± 15 µg/L to an influent concentration of 131 ± 51 µg/L. The ammonia concentration was consistently increased above the raw water concentration in F-NH_3_ A/B and F-Both A/B to an average concentration of 0.11 ± 0.05 mg/L. The dissolved oxygen concentration in the experimental filters was also increased above the raw water concentration to 7.1 ± 1.2 mg/L during the aeration period (Fig. 2a). Temperature for the experimental filters was comparable to the control filters.

### Removal of manganese, iron, and ammonia

**Figure 3:**
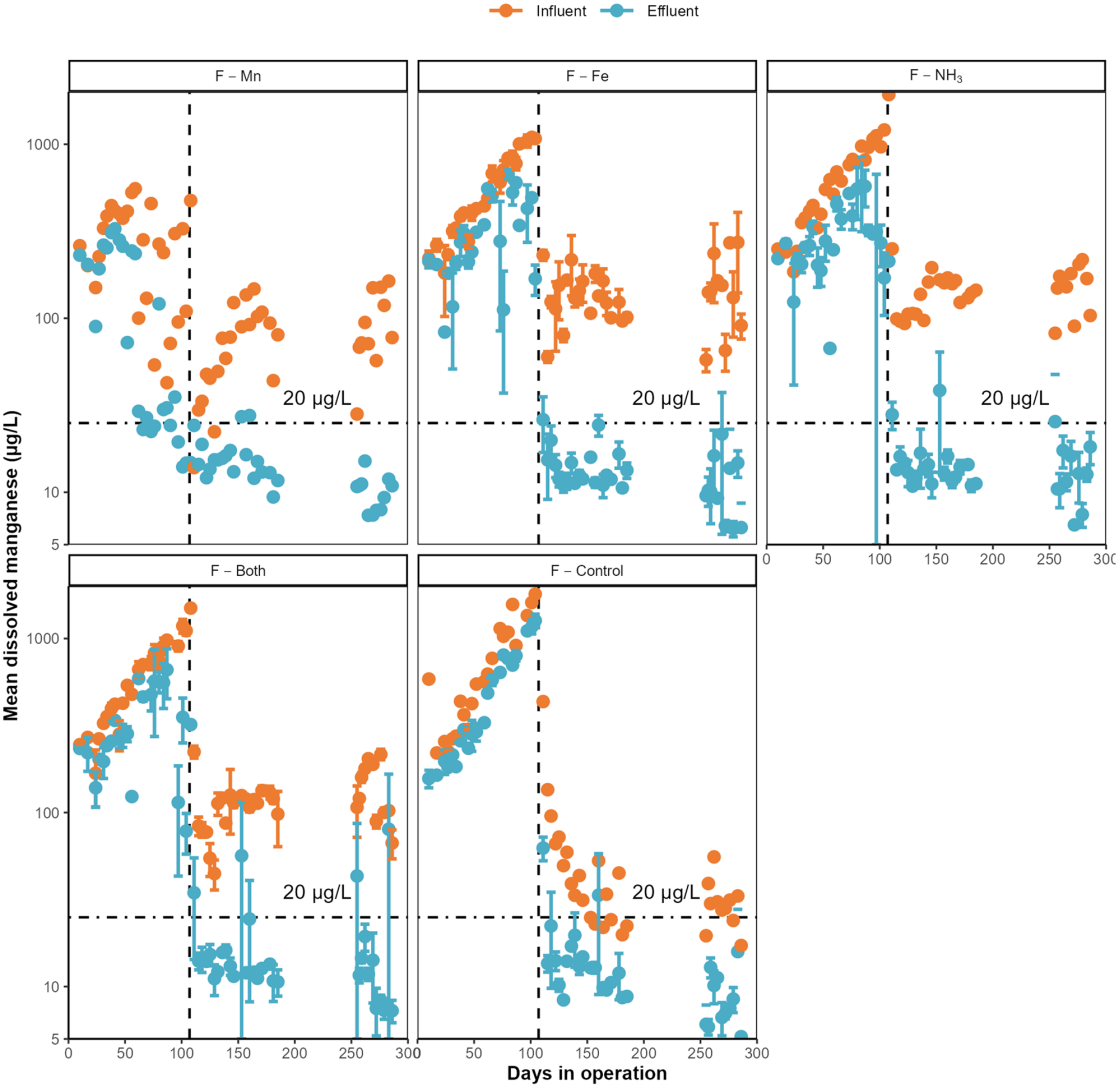
Mean dissolved manganese across raw water biofilters, grouped by influent condition. Error bars represent standard deviation. Vertical dashed lines represent the division between lake stratification and destratification.

Filter F-Mn was the first to approach the manganese treatment goal of 20 µg/L (Fig. 3). However, during this time this filter was unintentionally fed less manganese than the other experimental filters and cannot be directly compared to them. This reduction in influent manganese concentration was caused by the main water storage for F-Mn being aerated, compared to the mixing point being aerated in the other experimental filters. Aerating the water storage decreased the manganese concentration by allowing dissolved manganese to be removed by biological or heterogeneous removal mechanisms in the resevoir^10^. The remaining experimental filters all started to approach the manganese treatment goal 1–2 weeks prior to destratification, but only F-Mn achieved the goal during this time. The control filters were unable to remove more than 30% of the influent manganese while the lake was stratified (Supplementary Fig. S3). After Bennery Lake destratified on day 107, influent dissolved manganese concentrations reduced to 107 ± 58 µg/L and all filters typically had effluent manganese concentrations below 20 µg/L. Effluent concentrations appeared to approach a limit, between 6 and 10 µg/L. Operating the biofilters at a reduced flowrate for 64 days did not impact manganese removal performance for any conditions after the original flowrate was resumed. Based on this data, it is likely that excellent manganese removal from untreated water containing very high manganese and iron concentrations is possible using raw water biofiltration provided that there is sufficient dissolved oxygen.

**Figure 4:**
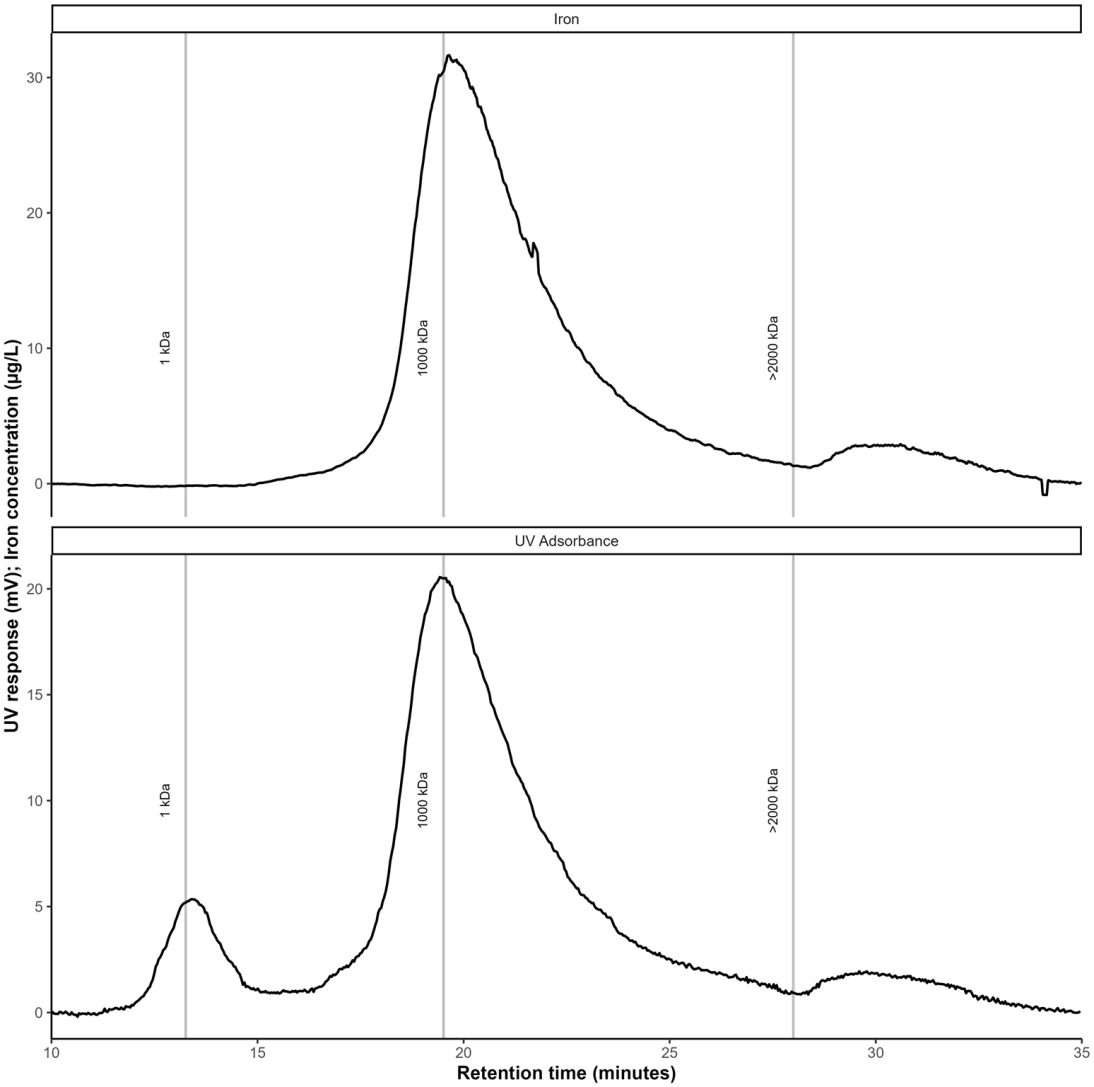
Iron fractogram for an untreated Bennery Lake sample taken on day 116.

Dissolved iron removal was moderate or poor for all filters, rarely exceeding 40% (Supplementary Fig. S3). At times the filters removed no iron or even released iron, particularly as influent iron concentrations reached their maximum values. After Bennery Lake destratified, effluent iron concentrations stabilized to a consistent level across all filters (Supplementary Fig. S4). This result was unexpected because iron is often easily removed from aerated water before it reaches the biofilter^27,28^. However, previous experiments at BLDWTP had similarly low iron removal^19^. It was hypothesized the iron in Bennery Lake is not truly dissolved, as iron typically is in groundwaters, and was instead partially composed of colloidal-sized particles which may be bound to organic matter. The size fractionation of influent iron was investigated using FFF and it was confirmed that a portion of the iron passing the 0.45 µm filter was not truly dissolved, and was observed in the range of 100–2000 kDa (Fig. 4). UV absorbance measurements at 254 nm also suggest that this fraction of the iron may be bound with organic matter, indicated by the overlapping peaks. These colloidal-sized particles do not appear to interact with the biofilter, indicating that the iron that is removed generally represents the truly dissolved portion. Fortunately, the iron that passes through this initial biofilter can be removed by coagulants applied before or after the dissolved manganese and iron are removed.

Ammonia removal was initially poor across F-NH_3_ A/B and F-Both A/B (0.11 ± 0.05 mg/L influent), despite only moderately increased concentrations over the raw water (0.05 ± 0.02 mg/L), with some filters even appearing to generate ammonia instead of removing it (Supplemental Fig. S5). Removal increased over time, with most filters achieving 50% or better ammonia removal 2 weeks before destratification and for 6 weeks after. After day 145 ammonia removal was inconsistent, with F-NH_3_ B performing best prior to the COVID interruption. After the COVID interruption ammonia removal was inconsistent across the filters but was typically greater than 40% one week after returning to the normal flowrate.

### Factors impacting manganese removal

It was expected that high levels of ammonia removal would be required before dissolved manganese removal could be achieved, based on groundwater biofilter studies which suggest that nitrifying bacteria may be responsible for manganese removal by providing adsorption sites, altering oxidation–reduction conditions, or by enabling the growth of manganese oxidizing bacteria^15,28^. However, effluent manganese concentrations below 20 µg/L were achieved despite inconsistent ammonia removal in the filters fed additional ammonia. The reduced impact of ammonia removal on manganese removal across these biofilters may be because the concentration of ammonia was lower than in other studies. For example, Ramsay et al.^28^ found that manganese removal did not occur prior to ammonium removal across groundwater biofilters with an influent ammonium concentration approximately double the concentration in this study (i.e., 0.2 mg/L). However, McCormick et al.^20^ suggest that nitrification across surface water biofilters with a comparable influent ammonia concentration (0.1 mg/L) was important for the development of manganese removal. Future investigations into the microbial community present in the biofilters may provide an explanation.

Aerating the biofilter influent water improved dissolved manganese removal when raw water dissolved oxygen concentrations were below 5 mg/L. This result was expected because the source of dissolved manganese was lake sediment exposed to anoxic water, and biological manganese removal is typically an aerobic process^29^. The experimental filters were aerated from day 63 to day 130, during which time their effluent dissolved manganese concentration was significantly lower than the control filters as a group (*p* = 0.007, Wilcoxon Rank Sum). Aeration triggered the onset of effective dissolved manganese removal across the experimental biofilters, with the entire group approaching complete removal even while manganese concentrations exceeded 1 mg/L, before the lake destratified (Fig. 3). The control filters were only able to effectively remove dissolved manganese after the lake destratified, and the dissolved oxygen concentration subsequently increased from 2.5 ± 0.8 mg/L to 8.1 ± 1.2 mg/L. The relationship between dissolved oxygen concentration and effluent dissolved manganese is supported by a moderate negative correlation across the entire dataset (ρ = − 0.58, Spearman).

Dissolved oxygen concentration is often neglected in surface water manganese treatment because dissolved manganese removal typically relies on strong oxidants or reactions catalyzed by free chlorine^6^. However, the significant impact of dissolved oxygen concentration on dissolved manganese removal observed in this study suggests that it deserves consideration. The manganese-oxide containing biofilms that are likely responsible for manganese removal across the raw water biofilters can accumulate at various locations in a conventional treatment train. In particular, the accumulation of these inorganic-biological systems is likely in transmission lines, on the surfaces of tanks, and in sludge blankets^6,30^. When dissolved oxygen is freely available, manganese-biofilms may remove dissolved manganese from water and accumulate manganese-oxides, providing an additional source of manganese removal capacity in the treatment system. However, if the dissolved oxygen concentration is depleted due to stratification or an unexpected oxygen demand, such as an algal bloom, manganese-biofilms may lose their removal capacity and/or may release dissolved Mn(II) into water past the point of control. Residual manganese oxides generated by chemical oxidation are also susceptible to the depletion of dissolved oxygen because manganese oxides are not stable in anoxic environments.

Previous studies have observed a negative correlation between manganese removal and iron loading (i.e., iron removed each filter run). For example, Bruins et al.^15^ found that filters with iron loadings above 2.7 kg Fe/m^2^ per filter run could not achieve complete manganese removal (> 80%), proposing that either iron-oxide minerals covered adsorption sites for dissolved manganese on filter media or that dissolved iron competed with dissolved manganese for adsorption sites. The biofilters in this study had extremely high iron loadings, sometimes exceeding 100 kg Fe/m^2^ per filter run when influent iron concentrations were highest (Supplementary Fig. S6). These high iron loadings did not inhibit dissolved manganese removal, with most filters achieving > 80% manganese removal after the initial acclimation period. This is the opposite of what Bruins et al.^15^ observed across over 100 groundwater biofilters, which may indicate that the mechanisms of manganese removal across surface water biofilters differ from groundwater biofilters. However, the limited impact of iron loading may also be due to differences in hydraulics and water chemistry in this study. For example, biofilters in this study were backwashed weekly, regardless of head loss, which could allow for the buildup of iron-oxides capable of dissolved manganese adsorption that would have been removed from a full-scale filter^15^.

A key difference between groundwater and surface water is the concentration and character of organic carbon, with the concentration and complexity of organic carbon typically higher in surface waters. In this study, the organic carbon concentration was moderate to high, ranging from 4.8 to 8.8 mg TOC/L, generally increasing for the duration of the experiment. Removal of TOC across the biofilters was poor, with a median value of 4.5% and a maximum removal of 22%. There was no correlation between TOC removal and effluent dissolved manganese (ρ = 0.07, Spearman), indicating that substantial organic carbon removal was not required to remove manganese from surface water. Additionally, the influent organic carbon concentrations increased over time, as did manganese removal, resulting in a positive correlation (ρ = 0.6, Spearman). This shows that manganese removal was not inhibited by the high concentrations of organic carbon found in surface waters.

**Figure 5:**
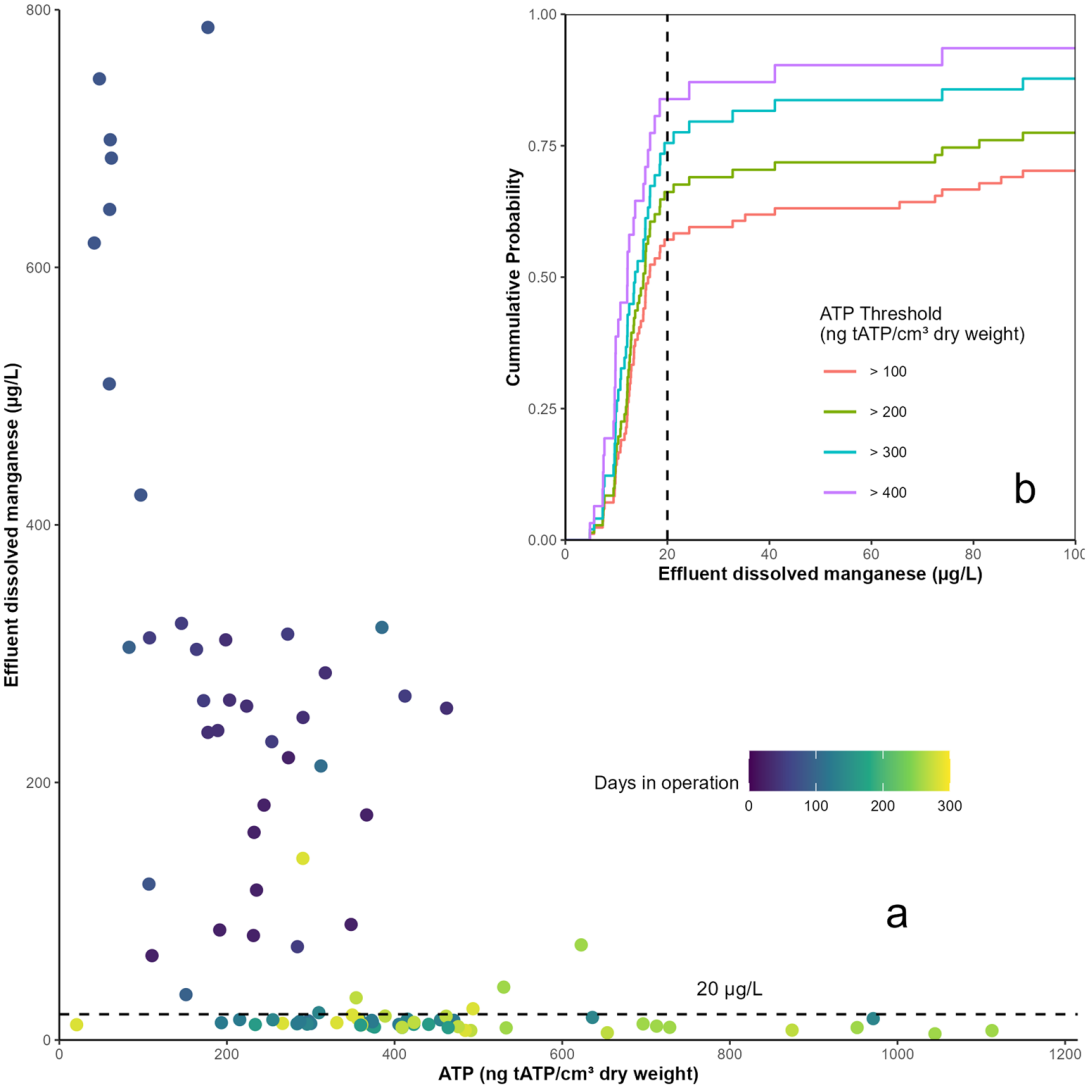
Relationship between ATP and effluent dissolved manganese (**a**) as a scatterplot with time and (**b**) as an empirical cumulative distribution function for manganese with multiple ATP values as thresholds.

ATP was quantified during the study to determine if there was a relationship between biomass quantity and manganese removal. Evans et al.^16^ found that manganese removal was generally poor when the ATP concentration was below 200 ng tATP/cm^3^ of dry media. The results of this study are similar, but manganese removal was not consistent above that threshold (Fig. 5a). Empirical cumulative distribution functions were calculated to evaluate possible ATP threshold values (Fig. 5b). When ATP was above 300 ng tATP/cm^3^, the probability of effluent dissolved manganese being below the guideline value of 20 µg/L was 0.75. Accordingly, effluent manganese removal was negatively correlated with ATP concentration (ρ = − 0.68, Spearman), suggesting that the general accumulation of biomass improved manganese removal. Other studies have shown that ATP concentrations are not usually related to filter performance, and instead are a general indication of filter maturity^31^. In-depth analysis of the microbial community within the biomass using DNA sequencing may provide further insight into the relationship between the biofilm and manganese removal.

Biofilters that remove manganese from water are known to accumulate manganese on filter media. This accumulation can lead to improved dissolved manganese removal performance, as manganese-oxides can adsorb and catalyze the oxidation of dissolved manganese^32^. In this study, biofilters began to accumulate manganese after approaching complete manganese removal (Supplementary Fig. S7). Any manganese removed prior to this did not significantly impact the quantity of manganese coating filter media. Manganese continued to accumulate over time, which caused a significant negative correlation between accumulated manganese and effluent dissolved manganese concentration (ρ = − 0.7, Spearman). Aluminum, calcium, and iron were also present in the filter media coating at high concentrations, but their concentrations were stable during the experiment and did not correlate with effluent dissolved manganese concentrations. This suggests that only manganese was incorporated into the biofilm and/or adhered to filter media and other metals were removed by periodic backwashing.

### Implications of this study

Water utilities have been utilizing basic aeration-biofiltration technologies to remove dissolved manganese from groundwaters for decades^29^. However, manganese in surface waters is usually controlled using either strong oxidants to oxidize and precipitate dissolved manganese or using chlorine and adsorptive media to adsorb and oxidize manganese^6^. Studies have demonstrated that surface water biofilters can be a viable treatment barrier for manganese^16,18,20^, but these studies considered conventional surface water biofiltration (i.e., pretreatment with chemical oxidation, coagulation, clarification, and biofiltration). The current study is distinct from previous surface water studies because it treated surface water using conventional groundwater biofiltration (i.e., aeration and biofiltration), and the results have implications for how the water industry may approach dissolved manganese control in surface water.

**Figure 6:**
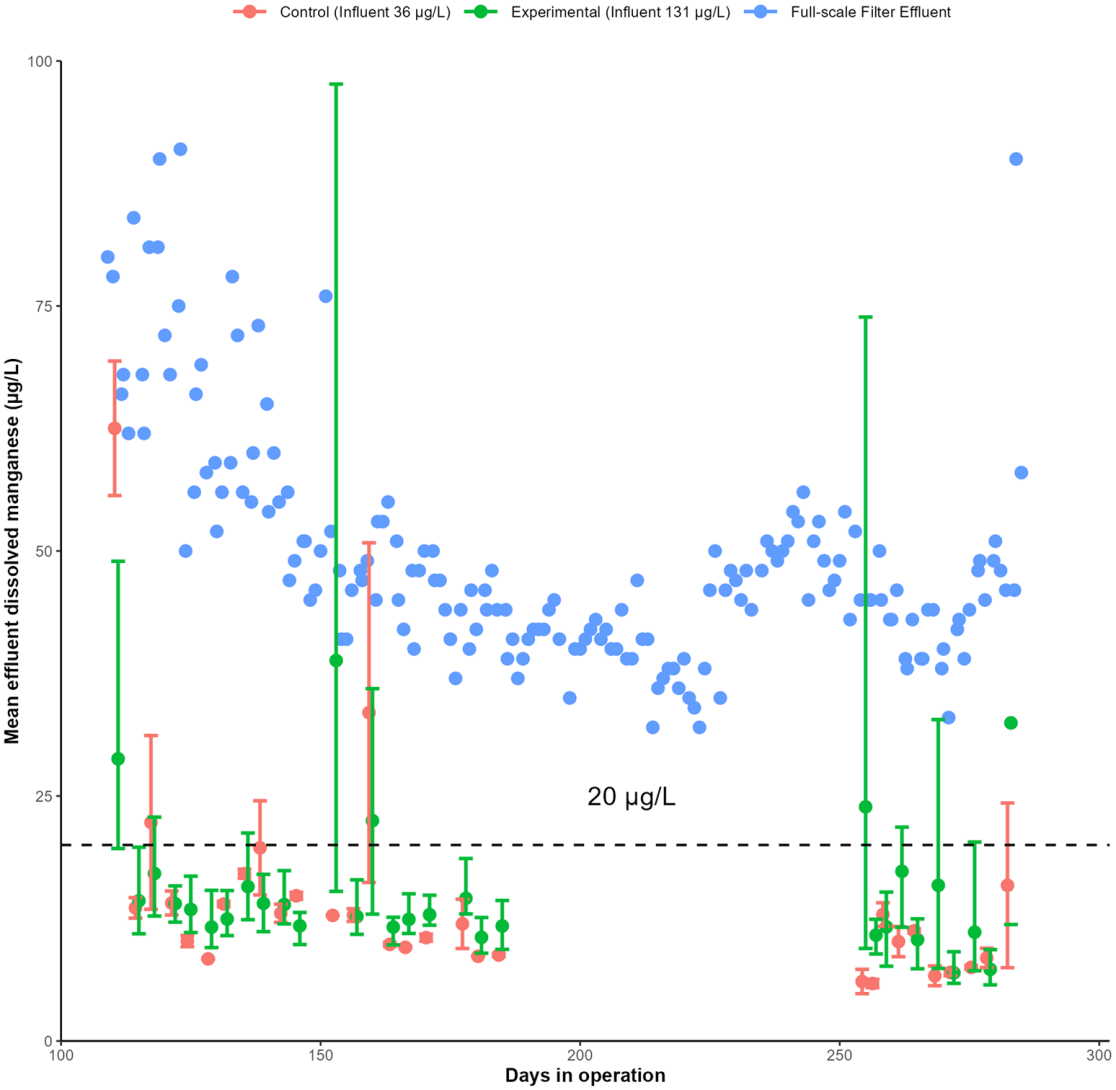
Effluent dissolved manganese for raw water biofilters and BLDWTP full-scale filter after destratification. Error bars represent maximum and minimum values. Concentration given in legend represent average influent manganese concentration during this period.

All aerated biofilters in this study were able to consistently remove > 80% of dissolved manganese after the initial acclimation period. This excellent removal resulted in a typical average effluent dissolved manganese concentration well below the Canadian aesthetic objective of 20 µg/L (Fig. 3), even when filters were supplied manganese concentrations exceeding 120 µg/L. The manganese removal performance of the raw water biofilters surpassed the permanganate driven full-scale process at BLDWTP (Fig. 6), despite the unadjusted influent manganese concentrations dropping to below 50 µg/L after destratification. BLDWTP relies on further removal of manganese by chlorine applied after filtration to achieve treatment goals. These results suggest that a simple raw water biofiltration system could be a viable method for controlling dissolved manganese from surface waters similar to the present study. Such a system could be implemented in regions without access to conventional manganese treatment technologies with minimal chemical inputs.

A unit process with the primary purpose of removing manganese may seem unnecessary to conventional surface water utilities, but it has precedent. Knocke et al.^33^ describes post-filtration contactors, which use free chlorine and media coated in manganese oxides to remove dissolved manganese passing through conventional treatment processes. This technology has since been used in full-scale treatment plants in Connecticut, Virginia, and Maine^34,35^. As the cost of oxidants increase, alternative manganese treatments such as these will become increasingly viable in surface water treatment plants. The raw water biofilters proposed here have the additional advantage of reducing the environmental impact of water treatment compared to post-filtration contactors, by not requiring free chlorine to remove manganese. However, the small anthracite and sand media used in the current study would cause head loss by particle retention and a corresponding need for frequent backwashing. This issue could be resolved using larger sized media, but significant work is required to prove the viability of this concept.

## Conclusions

- The results of this study demonstrate that a raw surface water biofiltration approach can be used to achieve effluent dissolved manganese concentrations below guideline values of 20 µg/L.
- High dissolved oxygen concentrations (> 8 mg/L) were critical in achieving low effluent dissolved manganese concentrations. Aeration also reduced the days in operation before manganese was effectively controlled.
- Poor ammonia removal (median 55%) and high iron loading (median 40 kg dissolved Fe/m^2^ per filter run) did not inhibit manganese removal, unlike what has been observed in groundwater studies.
- Raw water biofilters achieved lower effluent dissolved manganese concentrations than the conventional full-scale treatment process, demonstrating the potential for replacing conventional manganese treatments with biological technologies.
- The raw water biofiltration approach could be used to drinking water in regions that lack access to conventional manganese treatments. However, head loss and backwashing requirements may prevent adoption of this technology by conventional surface water treatment plants. We suggest that larger sized media could resolve this issue. However, substantial research in this area is required before such a technology can be adopted.

## Supplementary Information

Supplementary Information.

## Data Availability

The data that support the findings of this study are available from the corresponding author upon reasonable request.
