# Supplementary material for: Raw water biofiltration for surface water manganese control
Source: Sci Rep. 2023 Jun 3;13:9020. doi: 10.1038/s41598-023-36348-1 (PMC10239442; doi:10.1038/s41598-023-36348-1)
Supplement: Supplementary file 1 — Supplementary Information. [file 41598_2023_36348_MOESM1_ESM.pdf]

Raw Water Biofiltration for surface water manganese control

Martin R. Earle*[1], Amina K. Stoddart[1], Graham A. Gagnon[1]

2023-03-27

Supplemental Information

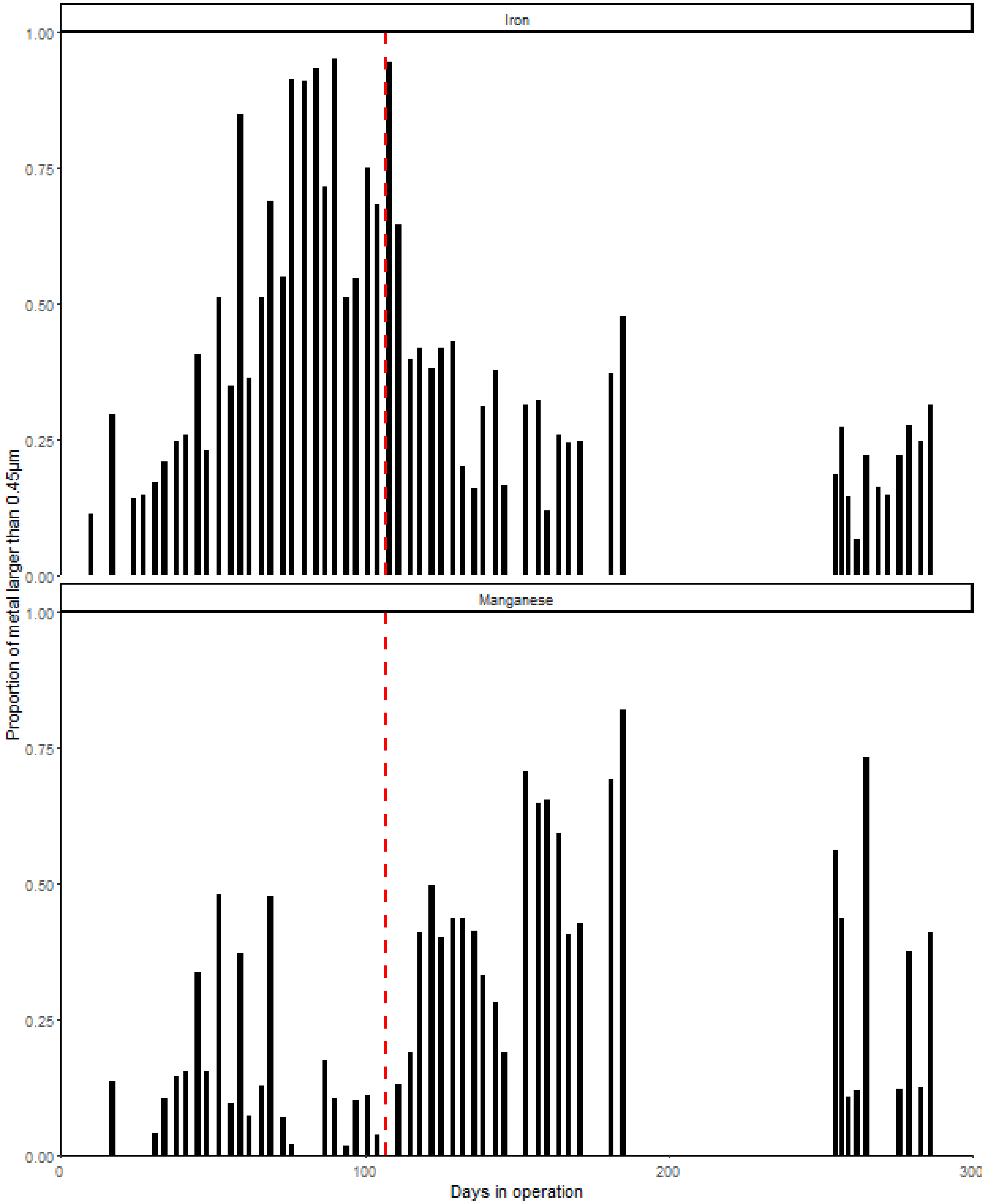

Figure S1: Proportion of particulate iron and manganese in raw water over time. Dashed line represents destratification.

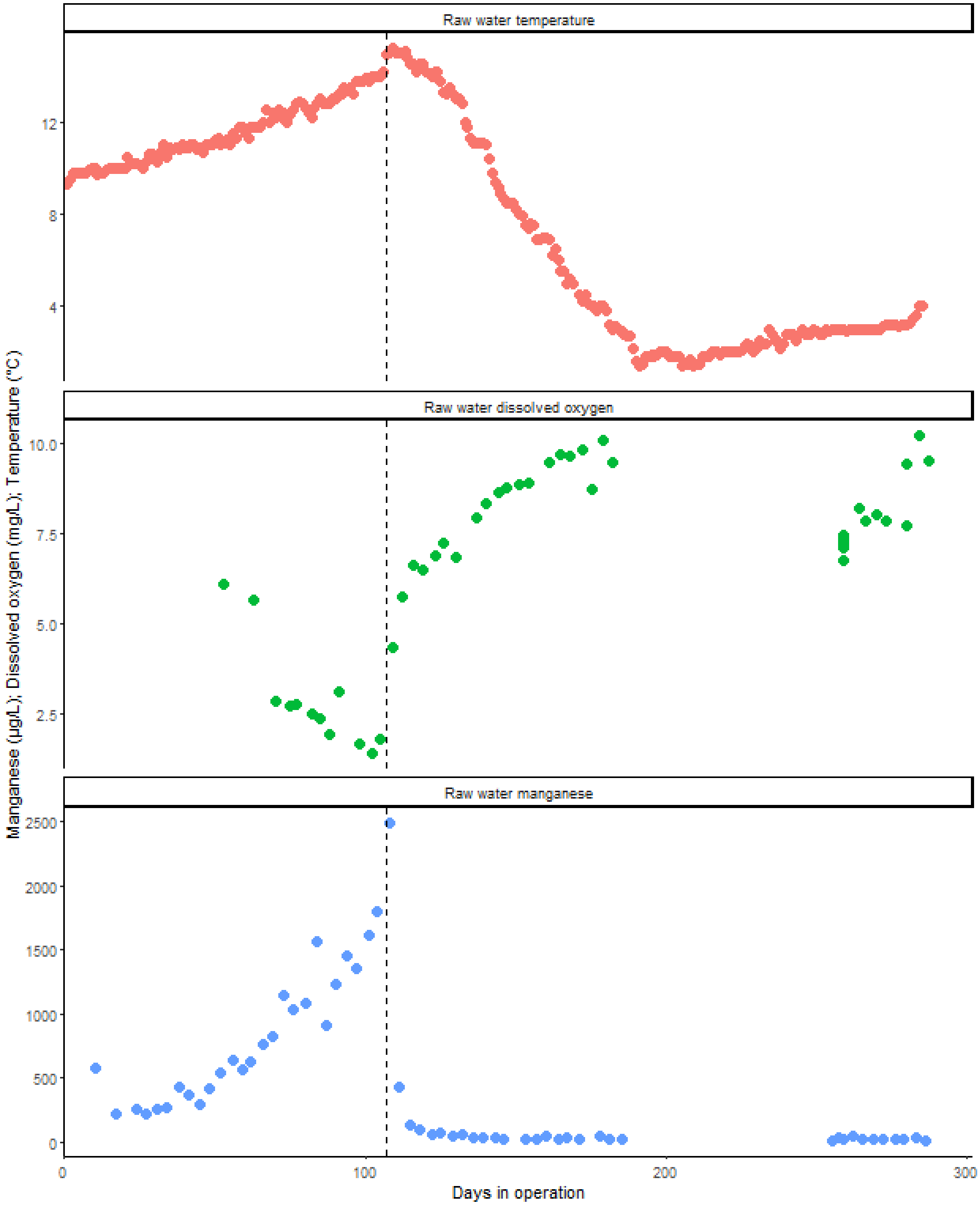

Figure S2: Raw water temperature, dissolved oxygen and manganese concentrations. Dashed line represents destratification.

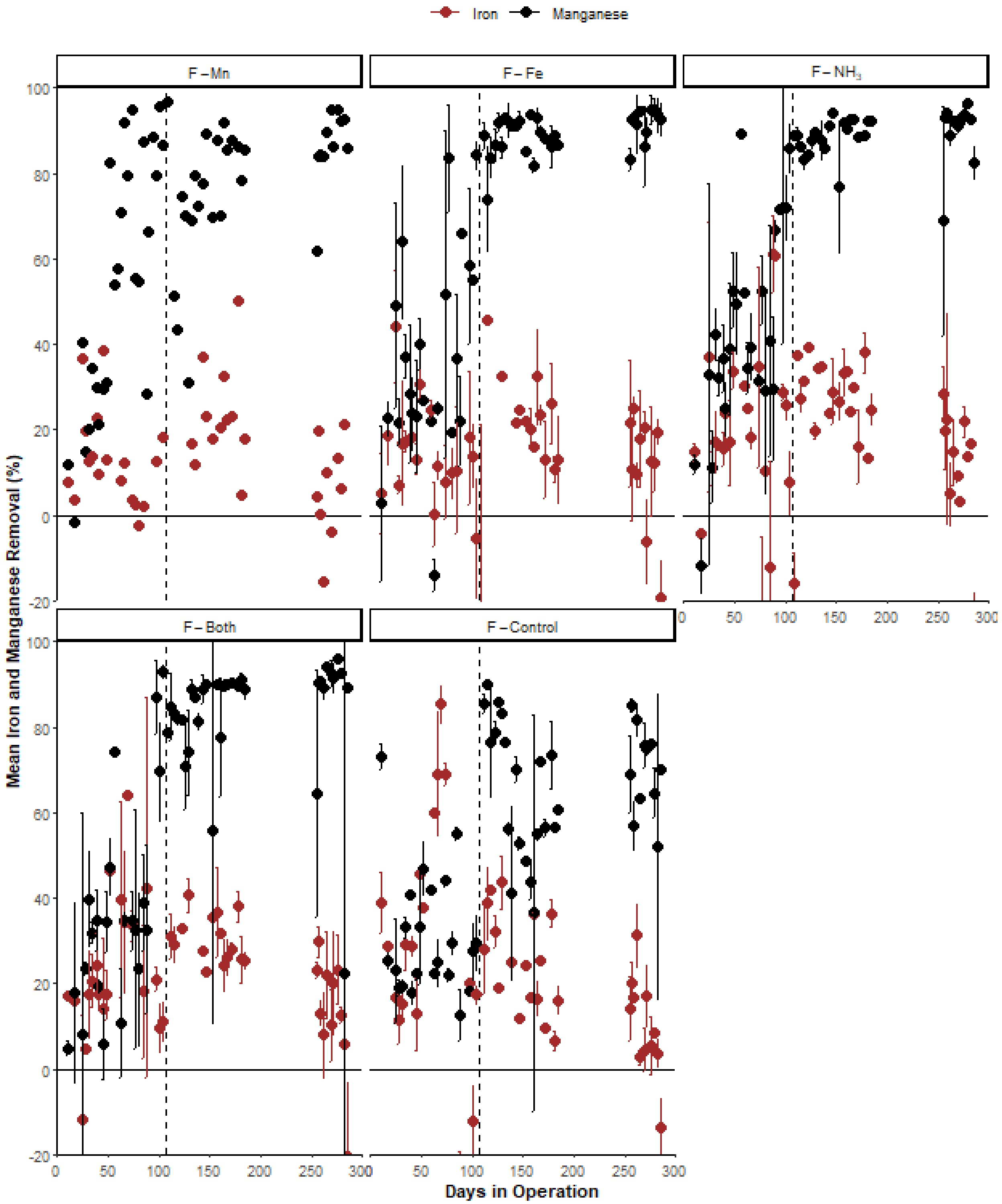

Figure S3: Mean iron and manganese removal across raw water biofilters, grouped by influent condition. Error bars represent standard deviation. Dashed lines represent the division between lake stratification and destratification.

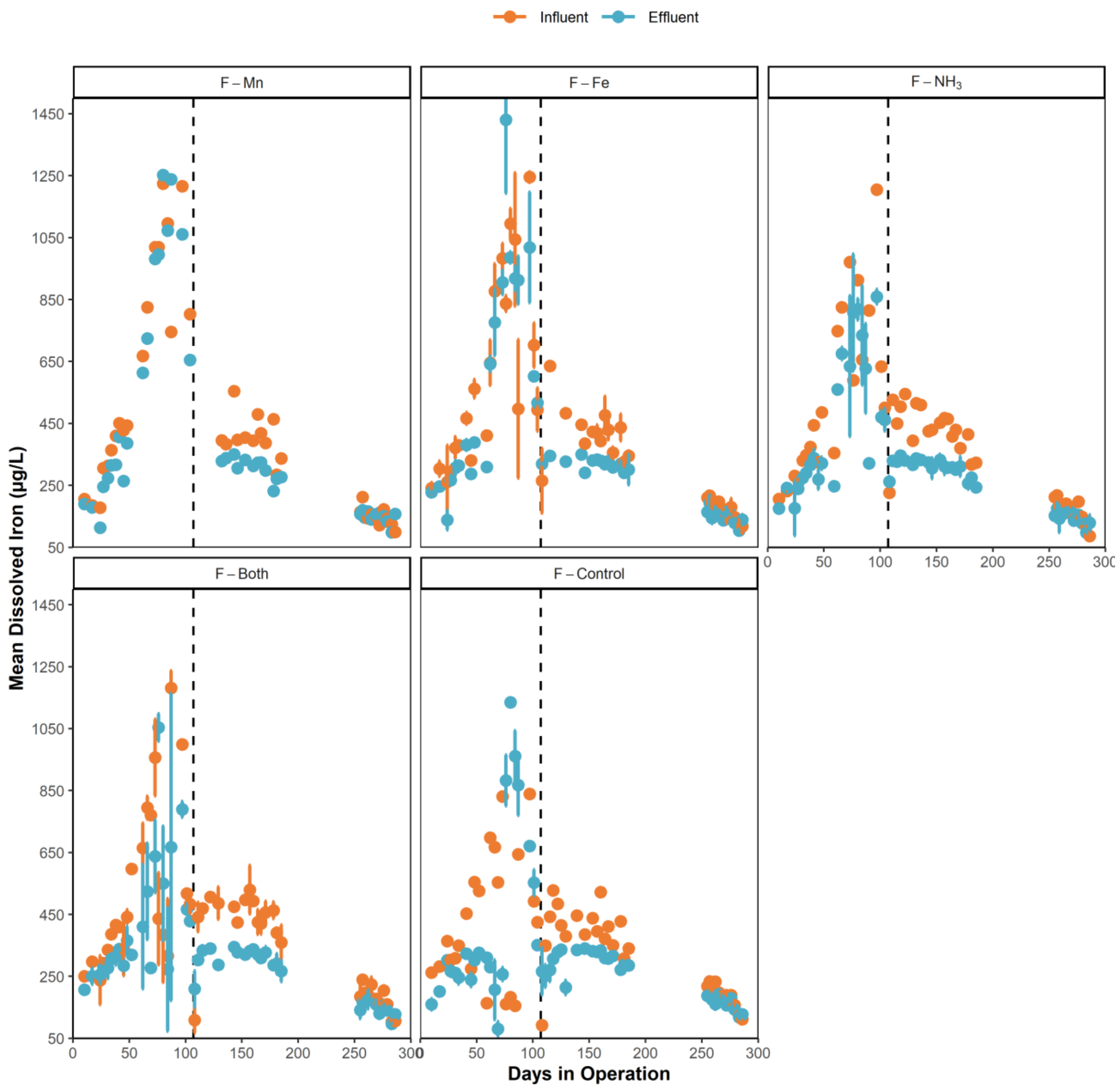

Figure S4: Mean effluent iron across raw water biofilters, grouped by influent condition. Error bars represent standard deviation. Dashed lines represent the division between lake stratification and destratification.

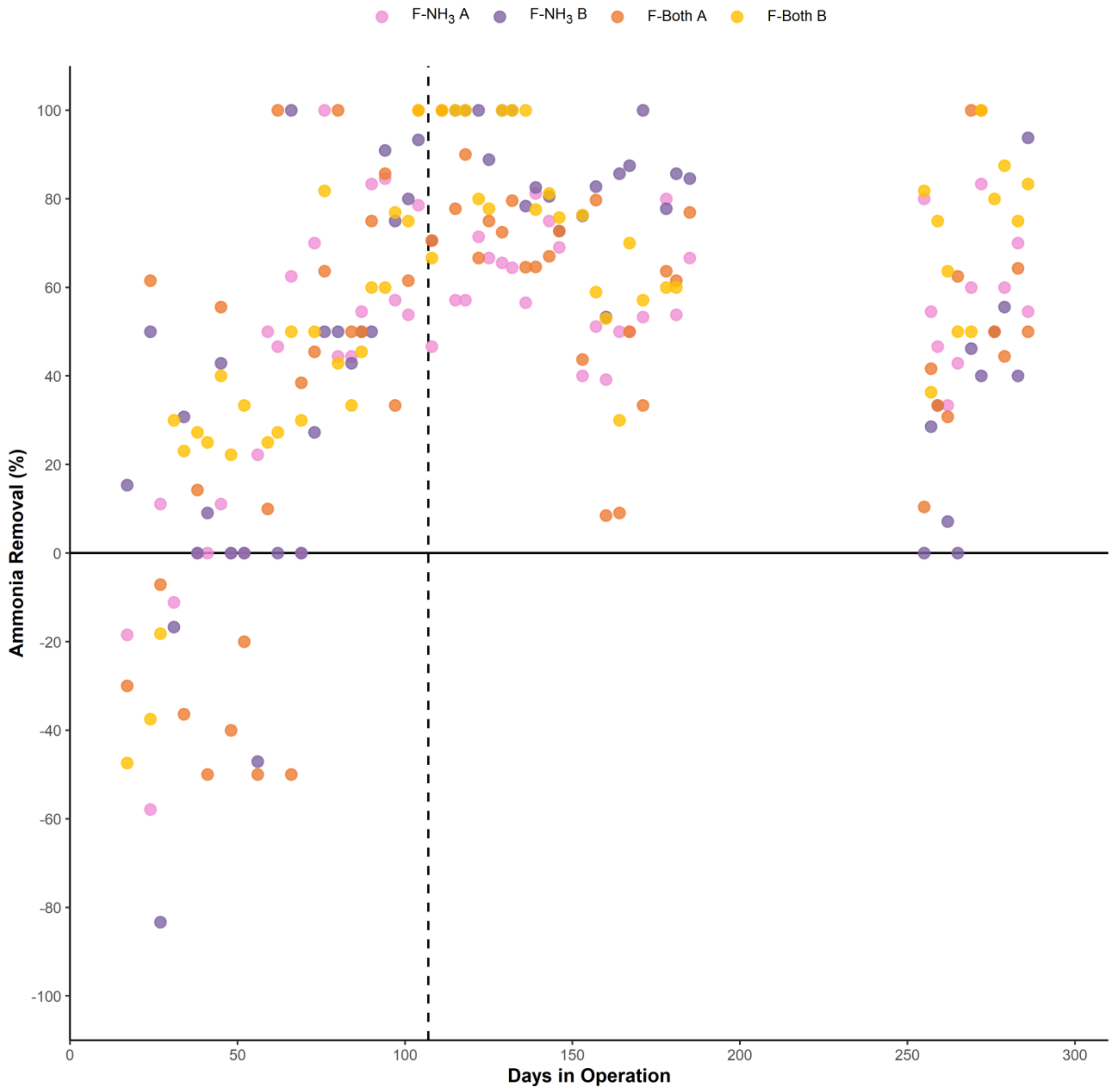

Figure S5: Ammonia removal for filters which were fed additional ammonia. The dashed line represents the division between lake stratification and destratification.

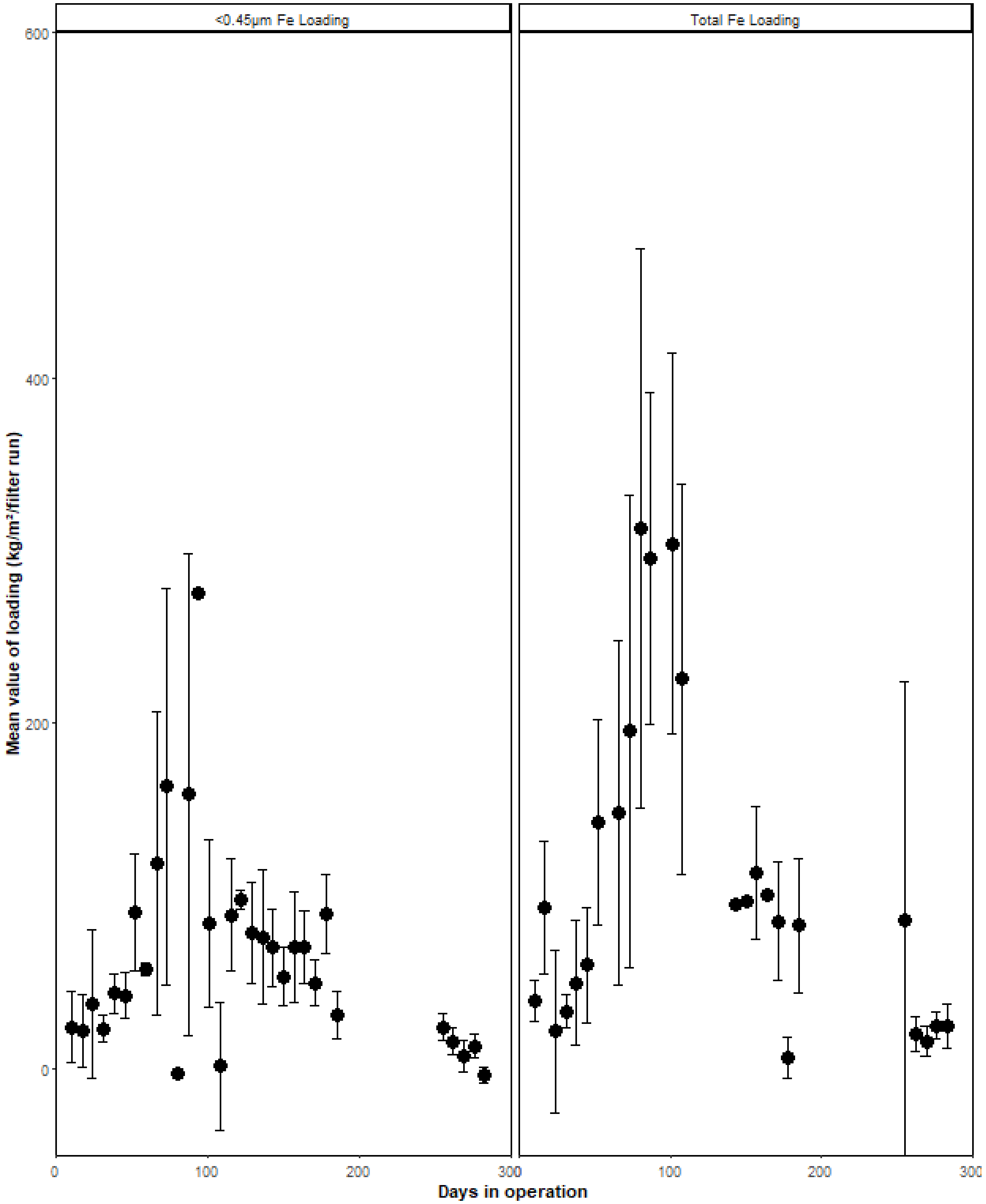

Figure S6: Mean iron loading by week. Error bars represent standard deviation.

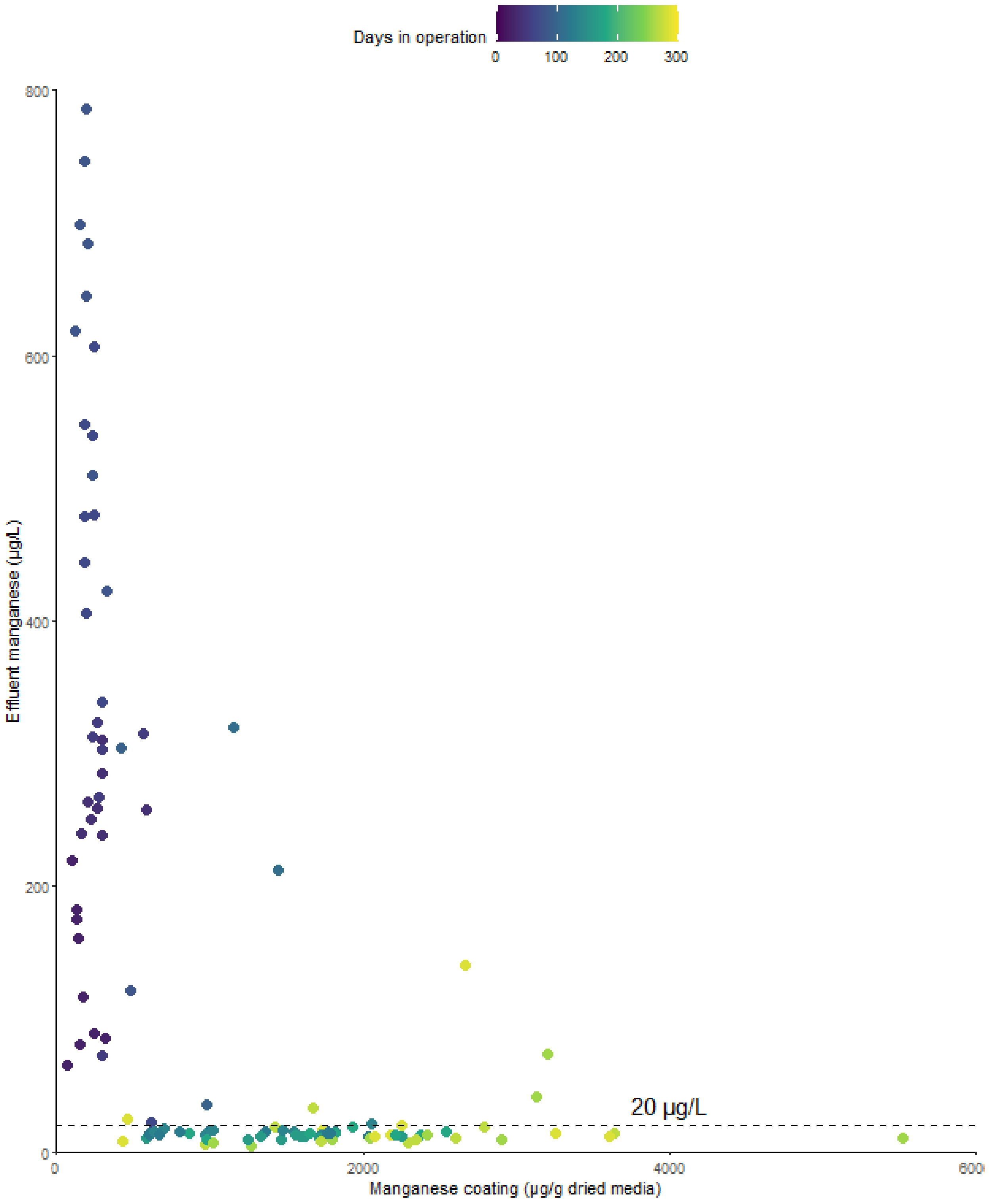

Figure S7: Manganese coating compared to effluent manganese concentration over time.
